# Characterization and Prediction of Protein Flexibility Based on Structural Alphabets

**DOI:** 10.1155/2016/4628025

**Published:** 2016-08-30

**Authors:** Qiwen Dong, Kai Wang, Bin Liu, Xuan Liu

**Affiliations:** ^1^Institute for Data Science and Engineering, East China Normal University, Shanghai 200062, China; ^2^College of Animal Science and Technology, Jilin Agricultural University, Changchun 130118, China; ^3^School of Computer Science and Technology, Harbin Institute of Technology Shenzhen Graduate School, Shenzhen 518055, China; ^4^College of Engineering, Shanghai Ocean University, Shanghai 201303, China

## Abstract

*Motivation.* To assist efforts in determining and exploring the functional properties of proteins, it is desirable to characterize and predict protein flexibilities.* Results.* In this study, the conformational entropy is used as an indicator of the protein flexibility. We first explore whether the conformational change can capture the protein flexibility. The well-defined decoy structures are converted into one-dimensional series of letters from a structural alphabet. Four different structure alphabets, including the secondary structure in 3-class and 8-class, the PB structure alphabet (16-letter), and the DW structure alphabet (28-letter), are investigated. The conformational entropy is then calculated from the structure alphabet letters. Some of the proteins show high correlation between the conformation entropy and the protein flexibility. We then predict the protein flexibility from basic amino acid sequence. The local structures are predicted by the dual-layer model and the conformational entropy of the predicted class distribution is then calculated. The results show that the conformational entropy is a good indicator of the protein flexibility, but false positives remain a problem. The DW structure alphabet performs the best, which means that more subtle local structures can be captured by large number of structure alphabet letters. Overall this study provides a simple and efficient method for the characterization and prediction of the protein flexibility.

## 1. Introduction

Proteins are dynamic molecules that are in constant motion. Their conformations are depending on environmental factors like temperature, pH, and interactions [1]. Some regions are more susceptible to change than others. Such motions play a critical role in many biological processes, such as protein-ligand binding [2], virtual screening [3], antigen-antibody interactions [4], protein-DNA binding [5], structure-based drug discovery [6], and fold recognition [7, 8].

Many studies try to predict protein flexibilities using either sequence or structure information of proteins [9]. Sonavane et al. [10] analyzed the local sequence features and the distribution of* B*-factor in different regions of protein three-dimensional structures. Yuan et al. [11] adopted support vector regression (SVR) approach with multiple sequence alignment as input to predict the* B*-factor distribution of a protein from its sequence. Schlessinger and Rost [12] found that flexible residues differ from regular and rigid residues in local features such as secondary structure, solvent accessibility, and amino acid preferences. They combined these local features and global evolution information for protein flexibility prediction. Several sequence-based* B*-factor prediction methods were compared by Radivojac et al. [13]. Different models have been proposed to predict* B*-factor distribution based on protein atomic coordinates. The normal mode analysis can identify the most mobile parts of the protein as well as their directions by focusing on a few C*α* atoms that move the most [14, 15]. The translation liberation screw model [16] simplified the protein as a rigid body with movement along translation, liberation, and screw axes. The Gaussian network model (GNM) [17] transformed a protein as an elastic network of C*α* atoms that fluctuate around their mean positions. Recently, Yang et al. [18] predicted the* B*-factor by combining local structure assembly variations with sequence-based and structure-based profiling. There are also many other methods for protein flexibility prediction [19–21].

All the above methods use the* B*- or temperature factors produced by X-ray crystallography to elucidate flexibilities of proteins. The* B*-factor reflects the degree of thermal motion and static disorder of an atom in a protein crystal structure [22]. However, there is noise in experimentally determinate* B*-factor. Many factors can affect the value of* B*-factor such as the overall resolution of the structure, crystal contacts, and, importantly, the particular refinement procedures [23].* B*-values from different structures can therefore not be reasonably compared [12]. Some researchers considered that the upper limit of accuracy for the prediction of* B*-factors is no more than 80% [11].

Protein structures are not static and rigid. The polypeptide backbones and especially the side chains are constantly moving due to thermal motion and the kinetic energy of the atoms (Brownian motion) [24]. Recent study [1] used the continuum prediction of secondary structures to identify the region undergoing conformational change. Other researchers have pointed out that continuous secondary structure assignment can capture protein flexibility [25]. Furthermore, the MolMovDB database [26] consists of structures that are experimentally determinate to exhibit conformational flexibility enabling a variety of protein motions. The Morph Server [27] in particular has been used by many scientists to analyze pairs of conformations and produce realistic animations.

The present work aims to explore whether the predicted conformations from the protein sequences can characterize their flexibilities or not. To achieve this goal, a simplified description of protein structure has to be provided first. The protein secondary structure offers only a summary of general backbone conformation and of local interactions through hydrogen bonding. The DSSP program [28] provides 8-class secondary structures. However, most secondary structures prediction methods only predict 3-class states with nearly 80% accuracy [29, 30]. The secondary structures are very crude description of protein backbone structures. Recently, many studies try to describe protein structures in a more refined manner. Toward this goal, many fragment libraries or structure alphabets (SA) have been presented either in Cartesian coordinates space or in torsion angles space [31–33]. Camproux et al. first derived a 12-letter alphabet of fragments by Hidden Markov Model [34] and then extended to 27 letters by Bayesian information criterion [35]. De Brevern et al. [36] proposed a 16-letter alphabet generated by a self-organizing map based on a dihedral angle similarity measure. The prediction accuracy of local three-dimensional structure has been steadily increased by taking sequence information and secondary structure information into consideration [37]. A comprehensive evaluation of these and other structural alphabets is performed by Karchin et al. [38].

In this study, we first explore whether the conformation variants can capture protein flexibility. The multiple conformations of proteins are taken from the Baker decoy sets [39]. Each three-dimensional conformation is represented by the one-dimensional series of letters from a structural alphabet. Four different structure alphabets, including the secondary structure in 3-class and 8-class, the PB structure alphabet [37], and the DW structure alphabet [40], are investigated here. Here, the conformational entropy is used to quantitatively indicate the flexibility. The results show that the conformational entropy has high correlation with* B*-factor. We then predict the protein flexibility from basic amino acid sequence. The structure alphabet letters of proteins are predicted using only sequence information and the entropy function of the predicted class distribution is used to be indicators of protein flexibilities. Experiment is performed on a subset of the MolMovDB database [26]. The results indicate that the conformational entropy is a good indicator of protein flexibility.

## 2. Materials and Method

### 2.1. Dataset

Three datasets are used in this study for different experimental validation.

The first dataset is taken from the work of Bodén and Bailey [1], which is used for the prediction of protein flexibility. This dataset contains 171 nonredundant protein sequences, in which no pair of sequences has larger than 20% sequence identity. All the proteins exhibit conformational flexibility according to the comprehensive database of macromolecular movements (MolMovDB) [26]. Each sequence in this dataset has been annotated with a list of residue positions that have more than one local structure according to the structure alphabets.

The second dataset is used to train the support vector machine which is used for the local structure predictions of proteins. This dataset is a subset of PDB database [41] obtained from the PISCES [42] web-server. There is less than 25% sequence identity between any two proteins and any protein has a resolution better than 2.5 Å. The structures with missing atoms and chain breaks are excluded. The proteins that show homologue with the proteins from the first dataset are also excluded. The resulting dataset contains 928 protein chains.

The third dataset is used to test whether the changes of local structures can characterize the protein flexibility. To achieve this goal, a variant of conformations for one protein must be provided. We use the Baker decoy sets [39] previously used for the evaluation of knowledge-based mean force potentials. This dataset consists of 41 single domain proteins with varying degrees of secondary structures and lengths from 25 to 87 residues. Each protein is attached with about 1400 decoy structures generated by ab initio protein structure prediction method of Rosetta [43].

### 2.2. Training and Test of Local Structures

Many methods have been presented for the prediction of protein local structures. The dual-layer model has been adopted here, which is developed in our previous studies [44]. The method is based on the observation that neighboring local structures are strongly correlated. A dual-layer model is then designed for protein local structure prediction. The position specific score matrix (PSSM), generated by PSI-BLAST [45], is inputted to the first-layer classifier, whose output is further enhanced by a second-layer classifier. At each layer, a variant of classifiers can be used, such as support vector machine (SVM) [33], neural network (NN) [46], Hidden Markov Models (HMM). In this study, the SVM is selected as the classifier, since its performance is better than those of other classifiers. Experimental results show that the dual-layer model provides an efficient method for protein local structure prediction.

### 2.3. Characterization of Protein Flexibilities by Conformational Changes

The conformations of proteins are represented by the local structures in the form of a structural alphabet. All the local structure types can be referred to as structure alphabet. Four different structure alphabets, including the secondary structure in 3-class and 8-class, the PB structure alphabet [37], and the DW structure alphabet [40], are investigated here. The three-dimensional protein structures can be represented by one-dimensional structure alphabet sequences according to a specific structure alphabet. Given a protein and its variable conformations, we can convert them into several structure alphabet sequences. The changes of local structures can be used to characterize the protein flexibility. For example, there is a protein sequence *a*_1_, *a*_2_,…, *a*_*n*_. Its three-dimensional structures and conformations are labeled as structure alphabet sequences; we then obtained a structure alphabet matrix *a*_11_, *a*_12_,…, *a*_*nm*_, where *a*_*ij*_ is the probability of the structure alphabet letter of the *j*th conformation at the amino acid position *i*, *n* is the length of the protein sequence, and *m* is the total number of letters in the structure alphabet. The conformational entropy is then used as an indicator of the protein flexibility:

(1) $$\begin{array}{c} H\left(\;i\;\right)=-\overset{m}{\underset{j=1}{\sum }}a_{ij}\ln a_{ij}, \end{array}$$

where *H*(*i*) is the conformational entropy of the protein at sequence position *i*.

The correlation between the conformational entropies and the *B*-factors is calculated as follows:

(2) $$\begin{array}{c} cc=\frac{\sum_{i=1}^{n}\left(H_{i}-Ave\left(H\right)\right)\left(B_{i}-Ave\left(B\right)\right)}{\sqrt{\left[\sum_{i=1}^{n}{\left(H_{i}-Ave\left(H\right)\right)}^{2}\right]\left[\sum_{i=1}^{n}{\left(B_{i}-Ave\left(B\right)\right)}^{2}\right]}}, \end{array}$$

where *B*_*i*_ is the* B*-factor of the protein at sequence position *i* and Ave(*H*) and Ave(*B*) are the average of the conformational entropy and the average of* B*-factor of the protein.

### 2.4. Prediction of Protein Flexibilities by Local Structure Entropies

Let the predicted local structure for a given residue be *Y* = *Y*_1_ *‚* … *‚Y*_*m*_ *‚* where *Y*_*j*_ is the probability that the residue is in the *j*th local structure class, and *m* is the number of local structure classes: 3 for 3-class secondary structure alphabet, 8 for 8-class secondary structure alphabet, 16 for PB structure alphabet, and 28 for DW structure alphabet. The conformation entropy of a residue is defined as

(3) $$\begin{array}{c} H_{}=-\overset{m}{\underset{j=1}{\sum }}Y_{j}\ln Y_{j}. \end{array}$$

High entropy indicates relative disorder. Low entropy indicates relative order.

### 2.5. Performance Metrics

The following measures are used to evaluate the prediction of protein flexibilities: sensitivity, specificity, precision, and the Receiver Operator Characteristic (ROC) curves, which are defined as follows:

(4) Sensitivity=TPTP+FN,Specificity=TNTN+FP,Precision=TPTP+FP,

where TP is the number of true positives (flexible residues correctly classified as flexible residues), FP is the number of false positives (rigid residues incorrectly classified as flexible residues), TN is the number of true negatives (rigid residues correctly classified as rigid residues), and FN is the number of false negatives (flexible residues incorrectly classified as rigid residues).

The ROC curve is plotted with true positives as a function of false positives for varying classification thresholds. A ROC score is the normalized area under the ROC curve. A score of 1 indicates the perfect separation of positive samples from negative samples, whereas a score of 0 denotes that none of the sequences selected by the algorithm is positive.

## 3. Results and Discussions

### 3.1. Local Structure Prediction

Four different structure alphabets are used in this study. They are the secondary structure in 3-class and 8-class, the PB structure alphabet [37], and the DW structure alphabet [40]. All of them are the description of the local structures of proteins.

The 3-class secondary structure provides a three-state description of backbone structures: helices, strands, and coils. The 8-class secondary structure provides a more detail description [28]. However, this description of protein structures is still very crude [47].

Two other structure alphabets are investigated in this study: the DW structure alphabet and the PB structure alphabet. They are represented in Cartesian coordinate space and in torsion angles space, respectively. The PB alphabet [37] is composed of 16 prototypes, each of which is 5-residue in length and represented by 8 dihedral angles. This structure alphabet remains valid although the size of the databank becomes large [48]. The DW structure alphabet is developed in our previous study [40], which is represented in Cartesian coordinate space. This structure alphabet contains 28 prototypes with lengths of 7 residues.

The dual-layer model is used to predict the local structures of proteins [44]. The experiment is performed on the second dataset. The *Q*-score is used to assess the prediction results, that is, the proportion of structure alphabet prototypes correctly predicted. This score is equivalent to the *Q*_3_ value for secondary structure prediction. After 5-fold cross-validation, the results are shown in Table 1. The accuracy of secondary structure prediction is comparable with the currently state-of-the-art method [29], while the performances of the other two structure alphabets are significantly better than those of other related works [33, 37, 49, 50]. For detailed results, please refer to Dong et al. [44].

### 3.2. Results for the Characterization of Protein Flexibilities

Since proteins are dynamic molecules, we can investigate whether the conformational changes can capture protein flexibilities. The protein structures are represented by structure alphabet sequences. The conformational entropy is used as an indicator of protein flexibility. The experiment is performed on the third dataset.

The initial results demonstrate that some of the proteins show high correlations between the conformational entropies and the* B*-factors while the other proteins show low and even negative correlations. After detail analysis, we find that the correlations are influenced by the distribution of the decoy structures. Uniform distribution often leads to high correlation. The decoy structures are first classified by the Root-Mean-Squared Deviation (RMSD) with the native structures. We then select the decoy structures so that they are approximately uniform distribution between different classes. Some of the proteins and the correlations and are listed at Table 2 together with the number of decoy structures. As the number of letters increases, the correlations also increase.

According to the law of thermodynamics, the native structure is the one that has the lowest energy. Since proteins are dynamically molecular in living organisms, their structures often fluctuate around the native state. The decoy sets used here are generated by the well-known Rosetta algorithm [43]. These sets contain many decoy structures whose energies are close to the native one. The conformational entropies are then derived from the decoy sets. Some of the conformational entropies show high correlation with the protein flexibilities. However, the decoy sets are not the true stories; there still are some proteins that show low correlations between the entropies and the* B*-factors (data not shown). This experiment only tries to investigate whether the conformational changes can capture protein flexibilities. If the true decoy sets can be obtained, we can give a definite answer. However, obtaining the true decoy sets is costly and labor-intensive work.

### 3.3. Results for the Prediction of Protein Flexibilities

The experiment is performed on the first dataset. Each residue is labeled as a rigid or flexible residue. The animations of protein motions provided by the MolMovDB database [26] are converted into structure alphabet letter sequences by the specific structure alphabet. If a residue changes its structure alphabet letter among the animations, it is labeled as flexible residue. Otherwise, it is labeled as rigid residue.

During the prediction process, the protein local structures are first predicted from amino acid sequence by the dual-layer model, and then the entropy function is applied to the predicted class distribution for each residue. Residues with entropy larger than a given threshold *T* are predicted to be flexible residues. Otherwise, they are predicted to be rigid residues. Following the work of Bodén and Bailey [1] we use the mean entropy of all residues in our conformation variability dataset as the threshold *T*.

**Figure 1:**
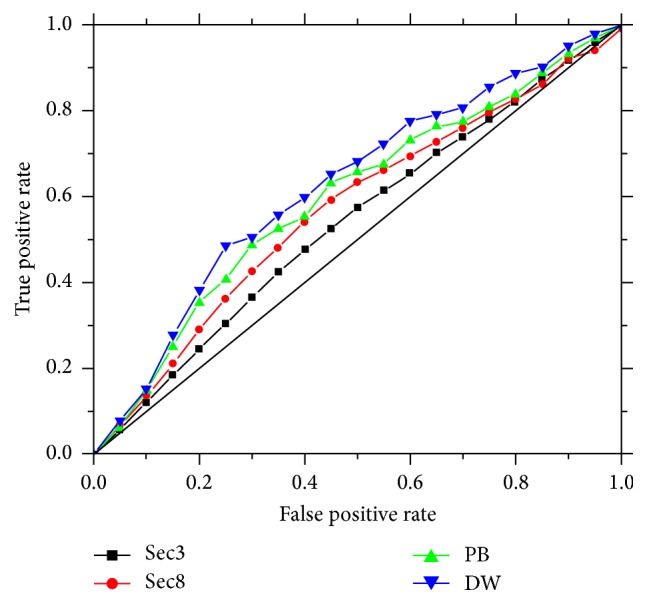
The ROC curve of the proposed method by using different structure alphabets on the set of 171 protein sequences.

The results of the four structure alphabets are shown in Table 3. The corresponding Receiver Operator Characteristic (ROC) curves are given at Figure 1. The different structure alphabets get different number of positive (flexible) and negative (rigid) samples. As the number of letters in the structure alphabet increases, the number of positive samples increases and the prediction performance also increases, which means that more subtle local structures can be captured by large number of structure alphabet letters. Particularly, the precision and ROC scores steadily increase. Overall the DW structure alphabet gets the best performance.

The results obtained here are similar to the work of Bodén and Bailey [1]. The precisions of this study are higher than that of Bodén and Bailey (0.05 for Sec3 and 0.12 for Sec8), but the ROC scores are a little lower than of Bodén and Bailey (0.61 for Sec3 and 0.64 for Sec8). The main differences of this study to that of Bodén and Bailey lie in two aspects. The first one is that the additional two structure alphabets (the PB and DW structure alphabet) are investigated here. The second one is that a decoy set is used to explore whether the conformation change can capture protein flexibility.

## 4. Conclusion

In this study we provide a simple and efficient method for the characterization and prediction of the protein flexibility. We first validate that the conformational change can capture protein flexibility and then predict protein flexibility from primary sequences. The results show that conformational entropy is a good indicator of protein flexibility. Four structure alphabets with different number of letters are investigated. Future work will aim at exploring other structure alphabets that can provide detail description of protein backbone structures and even the side-chain structures.

## Figures and Tables

**Table 1:** The average *Q*-scores of local structure prediction for the four structure alphabets.

	Sec3	Sec8	PB	DW
Number of letters	3	8	16	28

Single-layer model	0.756	0.593	0.564	0.432
Dual-layer model	0.765	0.614	0.585	0.456

The single-layer model uses the position specific score matrix (PSSM) as input and output probability of the structure alphabet letters. The dual-layer model adds an additional classifier, which uses the output of single-layer model as input and output final prediction. For both models, the support vector machine is used as the classifiers.

**Table 2:** The correlations between the conformational entropies and the *B*-factors.

ID	<3^a^	3-4	4-5	5-6	>6	Sec3^b^	Sec8	PB	DW
1res	73	73	73	7	4	0.1105	0.1505	0.2454	0.2605
1am3	571	177	162	161	400	0.1139	0.2993	0.4110	0.5149
1r69	389	119	284	228	300	0.2028	0.4040	0.3909	0.3794
1utg	1	20	401	290	300	0.2003	0.2990	0.2729	0.1653
1a32	364	125	95	142	300	0.2819	0.4818	0.5077	0.4145
1mzm	9	306	317	171	300	0.0118	0.2734	0.3353	0.3144
1hyp	1	0	34	270	300	0.1491	0.3579	0.1893	0.2889
1cei	1	0	4	64	300	0.0821	0.3583	0.4335	0.4932
1pgx	219	342	182	391	300	0.0264	0.2843	0.3339	0.3674
5icb	3	142	481	225	300	0.4255	0.5660	0.5433	0.5635
Ave	163.1	130.4	203.3	194.9	280.4	0.1604	0.3474	0.3663	0.3762

^a^Shown in the table are the numbers of decoy structures in this class.

^b^Shown in the table are the correlations measured by the specific structure alphabet.

**Table 3:** Prediction performance of the protein flexibilities by different structure alphabets.

SA^a^	No. po^b^	No. ne^c^	Sensitivity	Specificity	Precision	ROC
Sec3	6152	54737	0.6291	0.4543	0.1109	0.5457
Sec8	9468	51421	0.5887	0.5677	0.1942	0.5741
PB	10625	50264	0.6209	0.5521	0.2114	0.5901
DW	16012	44877	0.6399	0.5725	0.2586	0.6193

^a^The structure alphabet types.

^b^The number of positive samples (flexible residues).

^c^The number of negative samples (rigid residues).

## References

[1] Bodén M., Bailey T. L. (2006). Identifying sequence regions undergoing conformational change via predicted continuum secondary structure. *Bioinformatics*.

[2] Li J., Cai J., Su H. (2016). Effects of protein flexibility and active site water molecules on the prediction of sites of metabolism for cytochrome P450 2C19 substrates. *Molecular BioSystems*.

[3] Manoharan P., Chennoju K., Ghoshal N. (2015). Target specific proteochemometric model development for BACE1—protein flexibility and structural water are critical in virtual screening. *Molecular BioSystems*.

[4] Dunker A. K., Brown C. J., Lawson J. D., Iakoucheva L. M., Obradović Z. (2002). Intrinsic disorder and protein function. *Biochemistry*.

[5] Dyson H. J., Wright P. E. (2002). Coupling of folding and binding for unstructured proteins. *Current Opinion in Structural Biology*.

[6] Antunes D. A., Devaurs D., Kavraki L. E. (2015). Understanding the challenges of protein flexibility in drug design. *Expert Opinion on Drug Discovery*.

[7] Feng Z., Hu X. (2014). Recognition of 27-class protein folds by adding the interaction of segments and motif information. *BioMed Research International*.

[8] Chen J., Liu B., Huang D. (2016). Protein remote homology detection based on an ensemble learning approach. *BioMed Research International*.

[9] Petrovich A., Borne A., Uversky V. N., Xue B. (2015). Identifying similar patterns of structural flexibility in proteins by disorder prediction and dynamic programming. *International Journal of Molecular Sciences*.

[10] Sonavane S., Jaybhaye A. A., Jadhav A. G. (2013). Prediction of temperature factors from protein sequence. *Bioinformation*.

[11] Yuan Z., Bailey T. L., Teasdale R. D. (2005). Prediction of protein B-factor profiles. *Proteins: Structure, Function, and Bioinformatics*.

[12] Schlessinger A., Rost B. (2005). Protein flexibility and rigidity predicted from sequence. *Proteins: Structure, Function and Genetics*.

[13] Radivojac P., Obradovic Z., Smith D. K. (2004). Protein flexibility and intrinsic disorder. *Protein Science*.

[14] Alexandrov V., Lehnert U., Echols N., Milburn D., Engelman D., Gerstein M. (2005). Normal modes for predicting protein motions: a comprehensive database assessment and associated Web tool. *Protein Science*.

[15] Krebs W. G., Alexandrov V., Wilson C. A., Echols N., Yu H., Gerstein M. (2002). Normal mode analysis of macromolecular motions in a database framework: developing mode concentration as a useful classifying statistic. *Proteins: Structure, Function and Genetics*.

[16] Kuriyan J., Weis W. I. (1991). Rigid protein motion as a model for crystallographic temperature factors. *Proceedings of the National Academy of Sciences of the United States of America*.

[17] Haliloglu T., Bahar I., Erman B. (1997). Gaussian dynamics of folded proteins. *Physical Review Letters*.

[18] Yang J., Wang Y., Zhang Y. (2016). ResQ: an approach to unified estimation of *B*-factor and residue-specific error in protein structure prediction. *Journal of Molecular Biology*.

[19] Kovacs J. A., Chacón P., Abagyan R. (2004). Predictions of protein flexibility: first-order measures. *Proteins: Structure, Function and Genetics*.

[20] Xia K., Wei G. W. (2013). Stochastic model for protein flexibility analysis. *Physical Review E: Statistical, Nonlinear, and Soft Matter Physics*.

[21] Gu Y., Li D.-W., Brüschweiler R. (2015). Decoding the mobility and time scales of protein loops. *Journal of Chemical Theory and Computation*.

[22] Drenth J. (1994). *Principles of Protein Crystallography*.

[23] Tronrud D. E. (1996). Knowledge-based B-factor restraints for the refinement of proteins. *Journal of Applied Crystallography*.

[24] Sharma A., Manolakos E. S. (2015). Efficient multicriteria protein structure comparison on modern processor architectures. *BioMed Research International*.

[25] Andersen C. A. F., Palmer A. G., Brunak S., Rost B. (2002). Continuum secondary structure captures protein flexibility. *Structure*.

[26] Flores S., Echols N., Milburn D. (2006). The Database of Macromolecular Motions: new features added at the decade mark. *Nucleic Acids Research*.

[27] Krebs W. G., Gerstein M. (2000). The morph server: a standardized system for analyzing and visualizing macromolecular motions in a database framework. *Nucleic Acids Research*.

[28] Kabsch W., Sander C. (1983). Dictionary of protein secondary structure: pattern recognition of hydrogen-bonded and geometrical features. *Biopolymers*.

[29] Dor O., Zhou Y. (2007). Achieving 80% ten-fold cross-validated accuracy for secondary structure prediction by large-scale training. *Proteins: Structure, Function, and Bioinformatics*.

[30] Bondugula R., Xu D. (2007). MUPRED: a tool for bridging the gap between template based methods and sequence profile based methods for protein secondary structure prediction. *Proteins: Structure, Function, and Bioinformatics*.

[31] Kolodny R., Koehl P., Guibas L., Levitt M. (2002). Small libraries of protein fragments model native protein structures accurately. *Journal of Molecular Biology*.

[32] Holmes J. B., Tsai J. (2004). Some fundamental aspects of building protein structures from fragment libraries. *Protein Science*.

[33] Sander O., Sommer I., Lengauer T. (2006). Local protein structure prediction using discriminative models. *BMC Bioinformatics*.

[34] Camproux A. C., Tuffery P., Chevrolat J. P., Boisvieux J. F., Hazout S. (1999). Hidden Markov model approach for identifying the modular framework of the protein backbone. *Protein Engineering*.

[35] Camproux A. C., Gautier R., Tufféry P. (2004). A hidden Markov model derived structural alphabet for proteins. *Journal of Molecular Biology*.

[36] De Brevern A. G., Etchebest C., Hazout S. (2000). Bayesian probabilistic approach for predicting backbone structures in terms of protein blocks. *Proteins: Structure, Function and Genetics*.

[37] Etchebest C., Benros C., Hazout S., De Brevern A. G. (2005). A structural alphabet for local protein structures: improved prediction methods. *Proteins: Structure, Function, and Bioinformatics*.

[38] Karchin R., Cline M., Karplus K. (2004). Evaluation of local structure alphabets based on residue burial. *Proteins: Structure, Function and Genetics*.

[39] Tsai J., Bonneau R., Morozov A. V., Kuhlman B., Rohl C. A., Baker D. (2003). An improved protein decoy set for testing energy functions for protein structure prediction. *Proteins: Structure, Function, and Bioinformatics*.

[40] Dong Q.-W., Wang X.-L., Lin L. (2007). Methods for optimizing the structure alphabet sequences of proteins. *Computers in Biology and Medicine*.

[41] Kouranov A., Xie L., de la Cruz J. (2006). The RCSB PDB information portal for structural genomics. *Nucleic Acids Research*.

[42] Wang G., Dunbrack R. L. (2003). PISCES: a protein sequence culling server. *Bioinformatics*.

[43] Simons K. T., Bonneau R., Ruczinski I., Baker D. (1999). Ab initio protein structure prediction of CASP III targets using ROSETTA. *Proteins*.

[44] Dong Q., Wang X., Lin L., Wang Y. (2008). Analysis and prediction of protein local structure based on structure alphabets. *Proteins: Structure, Function, and Bioinformatics*.

[45] Altschul S. F., Madden T. L., Schäffer A. A. (1997). Gapped BLAST and PSI-BLAST: a new generation of protein database search programs. *Nucleic Acids Research*.

[46] Hawkins J., Bodén M. (2005). The applicability of recurrent neural networks for biological sequence analysis. *IEEE/ACM Transactions on Computational Biology and Bioinformatics*.

[47] Chu W., Ghahramani Z., Podtelezhnikov A., Wild D. L. (2006). Bayesian segmental models with multiple sequence alignment profiles for protein secondary structure and contact map prediction. *IEEE/ACM Transactions on Computational Biology and Bioinformatics*.

[48] de Brevern A. G. (2005). New assessment of a structural alphabet. *In Silico Biology*.

[49] Benros C., De Brevern A. G., Etchebest C., Hazout S. (2006). Assessing a novel approach for predicting local 3D protein structures from sequence. *Proteins: Structure, Function and Genetics*.

[50] Tang T., Xu J., Li M. (2005). Discovering sequence-structure motifs from protein segments and two applications. *Pacific Symposium on Biocomputing*.

